# Supplementary material for: Hepatic HuR modulates lipid homeostasis in response to high-fat diet
Source: Nat Commun. 2020 Jun 16;11:3067. doi: 10.1038/s41467-020-16918-x (PMC7298042; doi:10.1038/s41467-020-16918-x)
Supplement: Supplementary file 1 — Supplementary Information [file 41467_2020_16918_MOESM1_ESM.pdf]

Supplementary Information

Hepatic HuR modulates lipid homeostasis in response to high-fat diet

Zhang et al.

Supplementary Methods

Liver lipid metabolic analysis [provided by LipidALL Technologies Co., Ltd (Beijing, China)]

Lipid extraction: Lipids were extracted by using a modified version of the Bligh and Dyer's method [(1)]. Briefly, tissues were homogenized on a bead ruptor in 750 μL of chloroform:methanol 1:2 (v/v) with 10% deionized water, and then incubated for 30 min at 4 ℃. After that, 350 μL of deionized water and 250 μL of chloroform were added. The samples were then centrifuged and the lower organic phase containing lipids was pooled into a single tube and dried in a SpeedVac instrument. Samples were stored at -80 ℃ until analysis.

Lipid profiling: Polar lipids were analyzed using an Exion UPLC system coupled with a triple quadrupole/ion trap mass spectrometer (6500 Plus Qtrap; SCIEX) by LipidALL Technologies [(2-3)]. Separation of individual classes of polar lipids by normal phase (NP)-HPLC was carried out by using a Phenomenex Luna 3-μm silica column (internal diameter 150 × 2.0 mm) with the following conditions: mobile phase A (chloroform: methanol: ammonium hydroxide, 89.5:10:0.5) and mobile phase B (chloroform: methanol: ammonium hydroxide: water, 55:39:0.5:5.5). MRM transitions were set up for comparative analysis of various polar lipids. PC-14:0/14:0, PE-14:0/14:0, PS-d31-16:0/18:1, PA-17:0/17:0, PG-14:0/14:0, BMP-14:0/14:0, Cer-d18:1/15:0-d7, SM-d18:1/12:0, LPC-17:0, LPE-17:0, LPI-17:0, LPA-17:0, LPS-17:0 obtained from Avanti Polar Lipids (Alabaster, AL, USA) and PI-8:0/8:0 from Echelon Biosciences, Inc. (Salt Lake City, UT, USA) were used as internal standards. FFAs were quantitated using d3-FFA16:0 (Sigma-Aldrich) and d8-FFA20:4 (Cayman Chemicals). GM3s were quantitated by using d3-GM3 d18:1/18:0 from Matreya LLC. Glycerol lipids including diacylglycerols (DAG) and triacylglycerols (TAG) were quantified by using a modified version of reverse-phase HPLC/MRM [(4)]. Separation of neutral lipids was achieved on a Phenomenex Kinetex-C18 2.6-μm column (4.6 x 100 mm) using an isocratic mobile phase containing chloroform: methanol: 0.1 M ammonium acetate 100:100:4 (v/v/v) at a flow rate of 170 μL for 17 min. The levels of short-, medium-, and long-chain TAGs were calculated by referencing to spiked internal standards of TAG(14:0)3-d5, TAG(16:0)3-d5 and TAG(18:0)3-d5 obtained from CDN isotopes, respectively. DAGs were quantified by using d5-DAG16:0/16:0 and d5-DAG18:1/18:1 as internal standards (Avanti Polar Lipids).

Serum lipid metabolic analysis [provided by Shanghai Luming Biological Technology Co, LTD (Shanghai, China)]

Chemicals: All chemicals and solvents were analytical or HPLC grade. Water, methanol, acetonitrile and formic acid were purchased from CNW Technologies GmbH (Düsseldorf, Germany).

Sample Preparation: Serum (30 μL) dissolved in methanol containing internal standard and 300 μl of methanol: water (1:1 = v:v) was added to each sample. Trichloromethane (300 μL) was added to each aliquot, and the samples were dispersed by pipetting. Using an ultrasonic homogenizer to improve the reaction for 6 min at 500 w, all the mixtures of each sample were transferred to 1.5-mL tubes, then extracted by ultrasonication for 10 min in an ice water bath. The extract was centrifuged at 13000 rpm and 4 ℃ for 10 min, and lower organic phase containing liquid was dried in a freeze concentration centrifugal dryer. A mixture of methanol and trichloromethane (300 μL, 1/2, vol/vol) was added to each sample, whereupon samples were vortexed for 30 s, extracted by ultrasonication for 10 min in an ice water bath, and placed at 4℃ for 30 min. Samples were centrifuged at 13000 rpm, 4 ℃ for 15 min. The lower phase (300 μL) from each tube was dried in a freeze concentration centrifugal dryer, and the lipid residue was reconstituted with 200 μL of isopropanol-methanol (1:1, V/V). After vortexing for 30 s and sonicating for 3 min, the solution was transferred to a 1.5-mL centrifuge tube. After centrifugation at 12000 rpm, 4 °C for 15 min, 150 μL of supernatant was placed in an LC-MS vial for LC-MS analysis.

Introduction to chromatographic mass spectrometry methods: Instrument: Thermo Q-EXACTIVE. A Nexera UPLC (Shimadzu, Kyoto, Japan) system fitted with Q-Exactivequadrupole-Orbitrap mass spectrometer equipped with heated electrospqrayionization (HESI) source (Thermo Fisher Scientific, Waltham, MA, USA) was used to analyze the metabolic profile of both ESI-positive and ESI-negative ion modes. An ACQUITY UPLC BEH C18 column (1.7 μm, 2.1 × 100 mm) was employed in both positive and negative modes. The binary gradient elution system consisted of (A) acetonitrile-water = 6:4 (containing 0.1% formic acid,10 mmol/L Ammonium formate, v/v) and (B) acetonitrile-isopropanol = 1:9 (containing 0.1% formic acid,10 mmol/L Ammonium formate, v/v) and separation was achieved by using the following gradient: 30% B hold 3 min, 30–62% B over 3–5 min, 62–82% B over 5–15 min, 82 – 99% B over 15 – 16.5 min, the composition was held at 99% B for 1.5 min, then 18 – 18.1 min, 99% to 30% B, and 18.1 – 22 min holding at 30% B. The flow rate was 0.35 mL/min and the column temperature 45 ℃. All the samples were kept at 4 ℃ during the analysis. The injection volume was 5 μL. The mass range of the instrument was set at m/z 120-1800. The ion spray voltage was set at 3500 V in the positive ion mode and −3100 V in the negative ion mode. The heater temperature was set at 300 ℃ and the capillary temperature at 320 ℃. The Sheath Gas Flow rate was 45 arb, The Aux Gas Flow Rate was 15 arb and the Sweep Gas Flow

Rate was 1 arb. The S-Lens RF Level was 50%.

Data Preprocessing and Statistical Analysis: The acquired LC-MS raw data were analyzed by the Progenesis QI software (version 2.3, Waters Corporation，Milford, USA) using the following parameters. Precursor tolerance was set at 5 ppm, fragment tolerance was set at 5 ppm, and retention time (RT) tolerance was set at 0.02 min. Internal standard detection parameters were deselected for peak RT alignment, isotopic peaks were excluded for analysis, noise elimination level was set at 10.00, and minimum intensity was set to 15% of base peak intensity. The internal standards used were L-2- chlorophenylalanine and C-17.

Liver and serum energy metabolic analysis [provided by Shanghai Luming Biological Technology Co, LTD (Shanghai, China)]

High-resolution mass spectrometric analysis of serum and liver samples: Each mouse liver sample was processed separately into a homogenate in a 50% methanol solution at a mass-to-volume ratio of 1:10; a total of 50 samples were processed. 50 μL of the homogenate or 50 μL serum were added to 450 μL precipitant, in a 1:1 methanol: acetonitrile solution. The mixture was vortexed for 60 s and centrifuged at 18,000 × g and 4 °C for 10 min before 100 μL of the supernatant was transferred to vials for analysis. For analysis, chromatography was performed on an ACQUITY BEH C18 column (1.7 μm, 2.1 × 50 mm, Waters) using a DIONEX Ultimate 3000 UPLC system (Thermo Fisher Scientific). The mobile phase, consisting of solvent A (2 mM ammonium formate) and solvent B (acetonitrile), was delivered at a flow rate of 0.25 mL/min, using the following gradient: 5% B (0 min), 5% B (1.0 min), 60% B (5.0 min), 100% B (8.0 min), 100% B (11.0 min), 60% B (14.0 min), 5% B (15.0 min), and 5% B (18.0 min). The total analysis time for each sample was 18 min. Mass spectrometry was performed on a Q Exactivequadrupole-Orbitrap hybrid mass spectrometer (Thermo Fisher Scientific) under positive (+) and negative (−) ionization modes simultaneously on an ESI (±) ion source. The ion source parameters were set as follows: spray voltage, 2800 V; evaporation temperature, 350 ℃; sheath gas, 35 Arb; auxiliary gas, 10 Arb; capillary temperature, 350 ℃; and S-lens RF, 50. The compound parameters were set as follows: grade I, full scan, resolution 70,000; AGC target, 1e6; maximum TT, 100 ms; and scanning range, 70–1050 m/z. Propranolol and tolbutamide were used as positive and negative internal standard, respectively.

Abbreviation of the lipids

Abbreviations of the lipids

Lipids	abbreviations	Lipids	abbreviations
Triacylglycerol	TAG	Phosphatidylserine	PS
Diacylglycerol	DG	Lyso-PS	LPS
Monoglyceride	MG	Lyso-PC	LPC
Ceramide	CER	Lyso-PE	LPE
Sphingomyelin	SM	Lyso-PI	LPI
lysophosphatidic acid	LPA	Lyso-PA	LPA
Lysophosphatidylcholine	LPC	Free fatty acids	FFA
Lysophosphatidylethanolamine	LPE	Cholesteryl esters	CE
Lysophosphatidylinositol	LPI	Free cholesterols	Cho
phosphatidic acid	PA	Bis（monoacylglycerol）phosphate	BMP
Phosphatidylcholine	PC	Cardiolipins	CL
Phosphatidylethanolamine	PE	Sphingomyelins	SM
Phosphatidylglycerol	PG	Monosialo-dihexosyl gangliosides	GM3
Phosphatidylinositol	PI		

Supplementary Figures

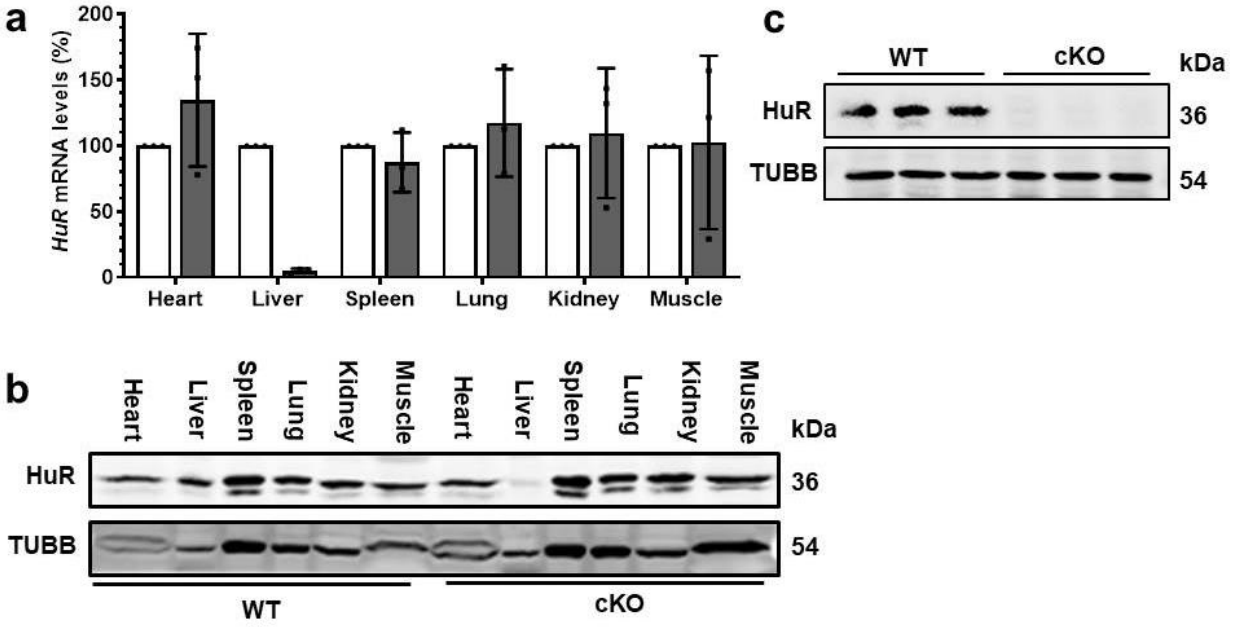

Supplementary Figure 1. Confirmation of hepatocyte-specific HuR knockout in mice. (a) RT-qPCR **(**Blank columns, WT; Black columns, cKO**)** was used to analyze *HuR* mRNA levels in tissues indicated. Data are the means ± SD from 3 repeated experiments **(one mouse tissues).** All the error bars are equivalent throughout the figure. **(b)** Western blot analysis (b) was used to analyze HuR protein levels in tissues indicated. Data are representative from 3 independent experiments. **(c)** Hepatocytes were isolated from livers of hepatocyte-specific HuR knockout (cKO, n=3) mice or wild-type (WT, n=3) littermates and HuR protein levels were tested by Western blot analysis to confirm the deletion of HuR in hepatocytes. Source data are provided as a Source Data file.

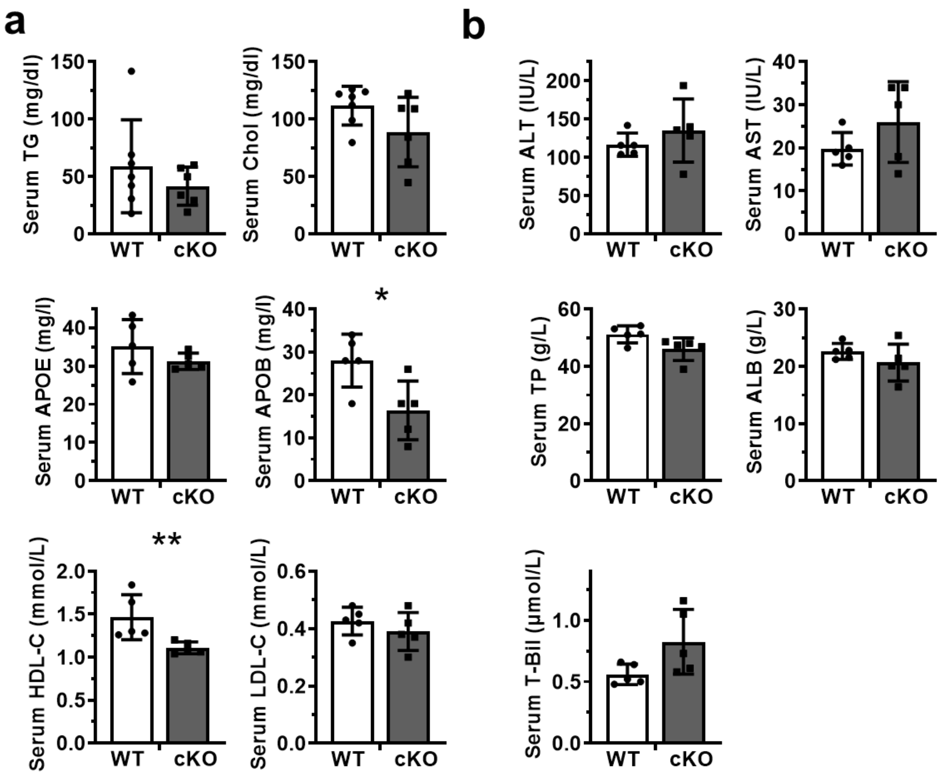

Supplementary Figure 2. Influence of hepatic HuR ablation on serum lipids and liver function. HuR cKO mice and WT littermates (N=5-7) were fed regular chow diet. **(a)** Levels of triglyceride (TG) (WT, n=7; cKO, n=6), cholesterol (Chol) (WT, n=7; cKO, n=6), APOB (WT, n=5; cKO, n=5), APOE (WT, n=5; cKO, n=5), high-density lipoprotein cholesterol (HDL-C) (WT, n=5; cKO, n=5), and low-density lipoprotein cholesterol (LDL-C) (WT, n=5; cKO, n=5) in serum. **(b)** Levels of alanine aminotransferase (ALT) (WT, n=5; cKO, n=5), aspartate aminotransferase (AST) (WT, n=5; cKO, n=5), total bilirubin (T-Bil) (WT, n=5; cKO, n=5), albumin (ALB) (WT, n=5; cKO, n=5), and total protein (TP) (WT, n=5; cKO, n=5) in serum. Data are the means ± SD; significance was analyzed by using two-tailed Mann-Whitney *U* test (*, $p < 0.05$; **, $p < 0.01$). All the error bars are equivalent throughout the figure. Source data are provided as a Source Data file.

a

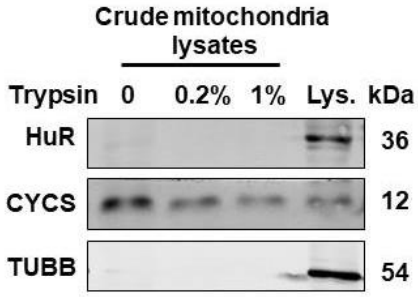

b

Potential targets of HuR (Mouse ATP synthesis)

Ndufb11	Atp6v0c	NDUFB6	Sdha
Atp6v1h	Cox6a1	Atp6ap1	Ndufa13
Atp5a1	Cox7a2	Sdhc	Ndufb8
Atp6v1a	Ndufa4	Cox7b	UQCRB
Atp6v1b2	Cox15	Atp6v1g1	Atp6v1d
Atp6v1e1	Ndufs1	Ndufa7	Ndufc2
Atp6v0e	Atp5g3	Uqcrh	

Supplementary Figure 3. Absence of HuR in mitochondria and predicted HuR target mRNAs encoding factors implicated in ATP synthesis. (a) Crude mitochondria lysates were treated with trypsin at the doses indicated, whereupon the presence of HuR, CYCS, and TUBB in the mitochondria (and control lysate, 'Lys.') was analyzed by Western blot analysis. Data are representative from 3 independent experiments. Source data are provided as a Source Data file. **(b)** List of mRNAs predicted bioinformatically to be HuR targets, encoding proteins implicated in ATP synthesis. All targets were obtained from reported Clip-seq data and analyzed by starBase v2.0 (http:/starbase.sysu.edu.cn/index.php46) [5].

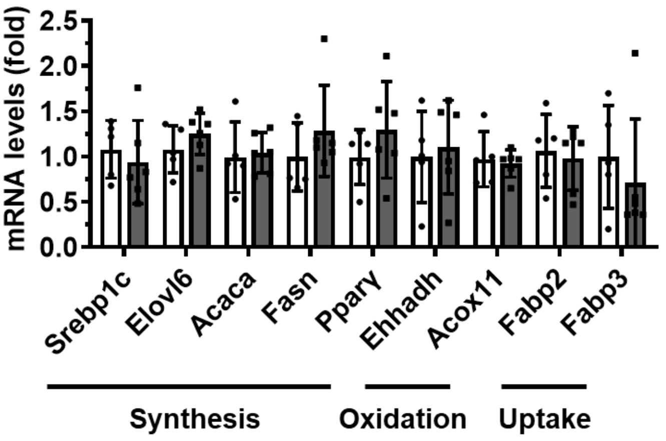

Supplementary Figure 4. Effect of HuR deletion on the levels of mRNAs encoding factors involved in lipid synthesis, oxidation, and uptake. RNA prepared from livers described in Figure 3 was subjected to RT-qPCR analysis to determine the levels of mRNAs encoding the factors indicated. Data are the means ± SD from 5-6 mice (WT, n=5; cKO, n=6). Blank columns, WT; Black columns, cKO. All the error bars are equivalent throughout the figure. Source data are provided as a Source Data file.

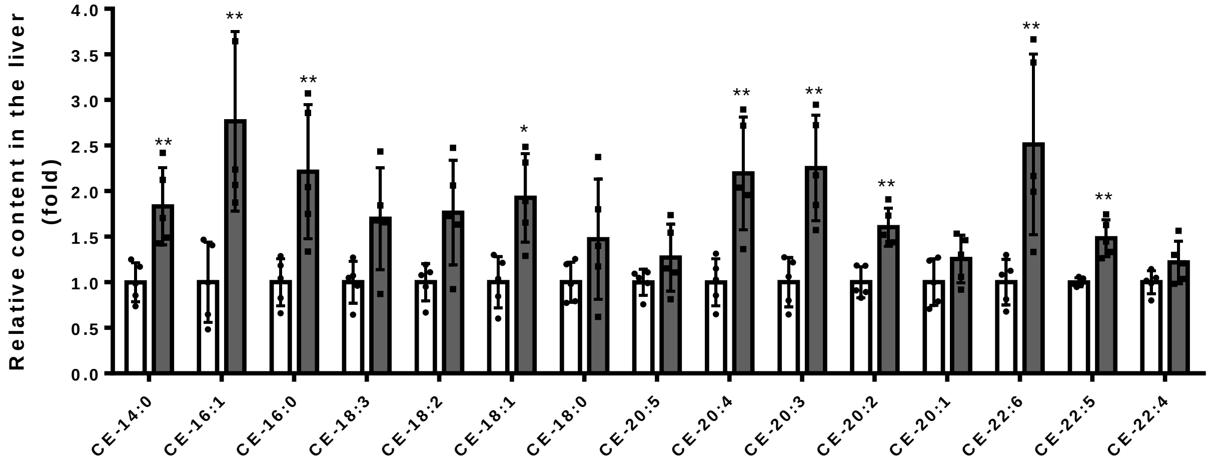

Supplementary Figure 5. Lipid metabolomic analysis in liver was analyzed. The relative amounts of CEs indicated are presented as the means ± SD from 5 mice (WT, n=5; cKO, n=5); significance were analyzed by using two-tailed Mann-Whitney *U* test (*, $p < 0.05$; **, $p < 0.01$). All the error bars are equivalent throughout the figure. Source data are provided as a Source Data file. The raw data can be found in the Supplementary Dataset 1.

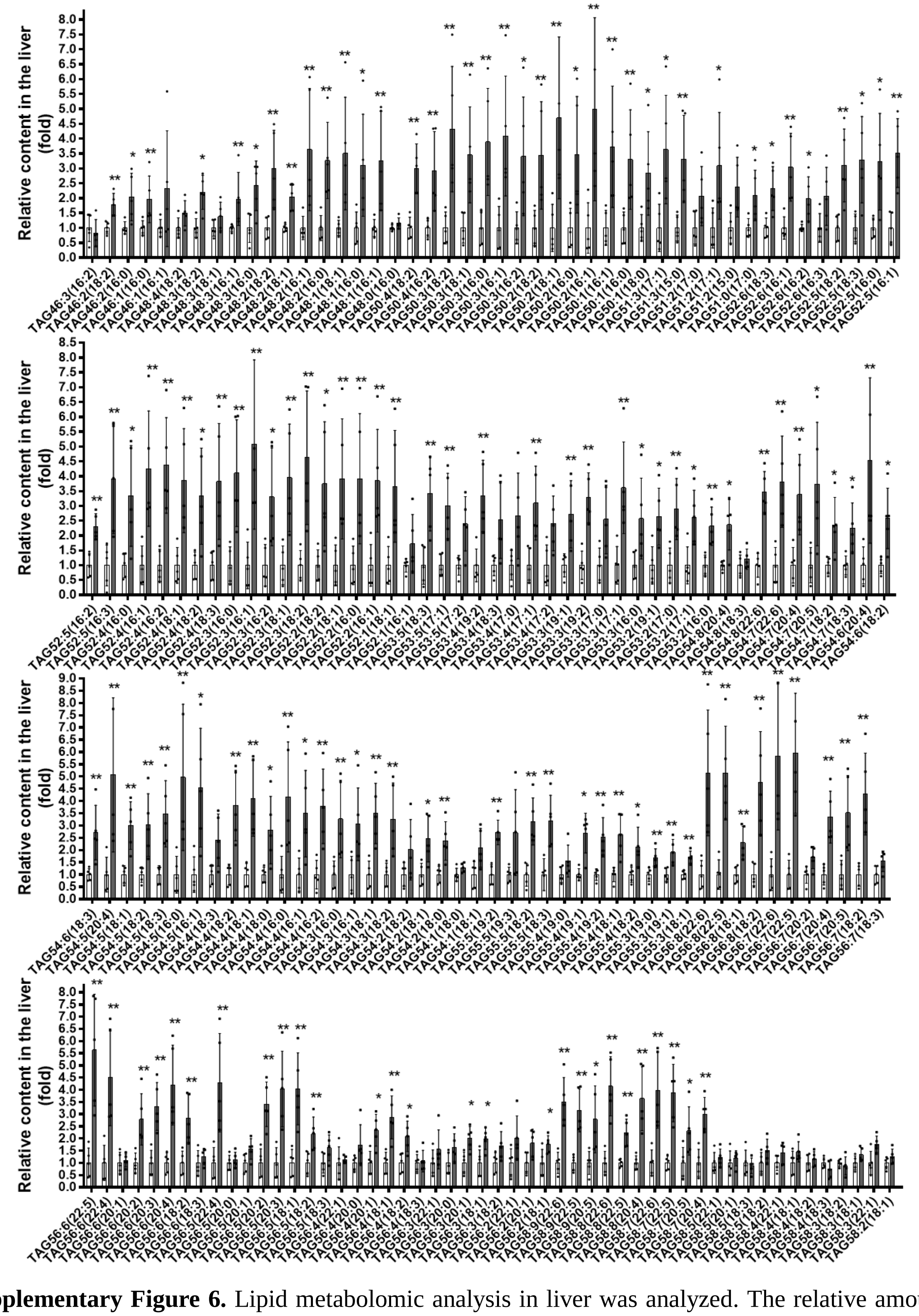

Supplementary Figure 6. Lipid metabolomic analysis in liver was analyzed. The relative amounts of the TAGs indicated are presented as the means ± SD from 5 mice (WT, n=5; cKO, n=5); significance were analyzed by using two-tailed Mann-Whitney *U* test (*, $p < 0.05$; **, $p < 0.01$). All the error bars are equivalent throughout the figure. Source data are provided as a Source Data file. The raw data can be found in the Supplementary Dataset 1.

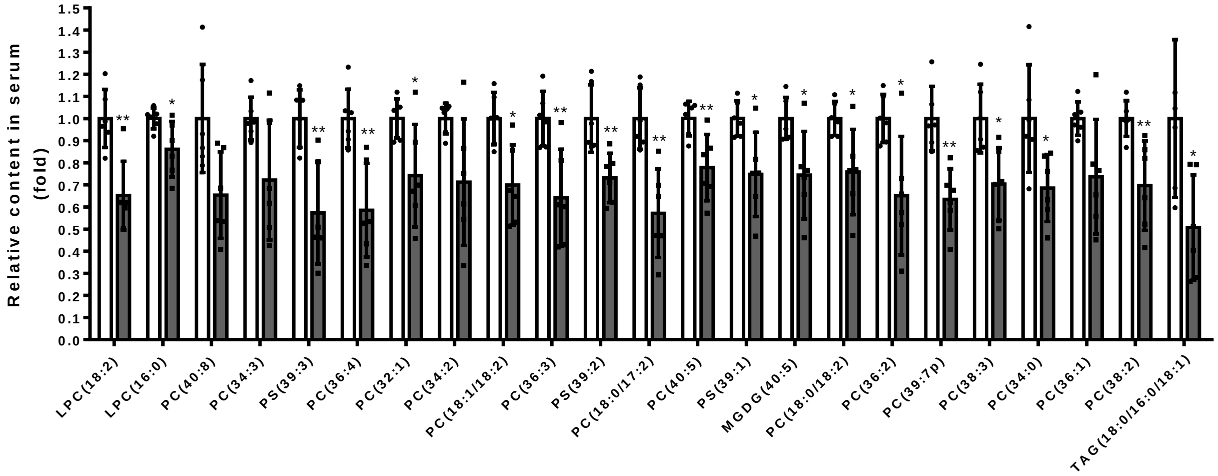

Supplementary Figure 7. Lipid metabolomic analysis in serum was analyzed. The relative amounts of the lipids indicated are presented as the means ± SD from 6 mice (WT, n=6; cKO, n=6); significance were analyzed by using two-tailed Mann-Whitney *U* test (*, $p < 0.05$; **, $p < 0.01$). All the error bars are equivalent throughout the figure. Source data are provided as a Source Data file. The raw data can be found in the Supplementary Dataset 1.

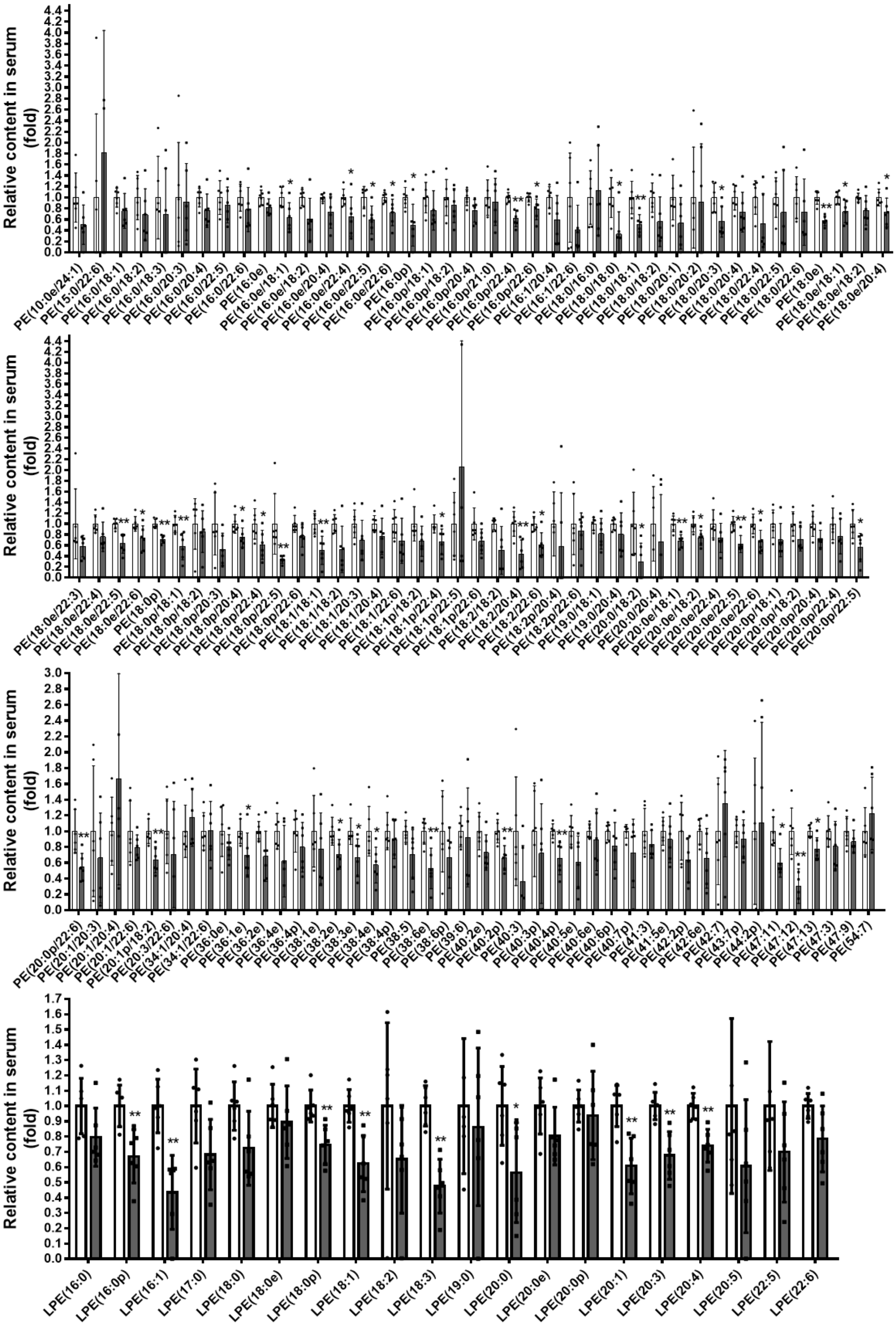

Supplementary Figure 8. Lipid metabolomics in serum was analyzed. The relative amounts of the lipids (PEs and LPEs) are presented as the means ± SD from 6 mice (WT, n-6; cKO, n=6); significance were analyzed by using two-tailed Mann-Whitney *U* test (*, p < 0.05; **, p < 0.01). All the error bars are equivalent throughout the figure. Source data are provided as a Source Data file. The raw data can be found in the Supplementary Dataset 1.

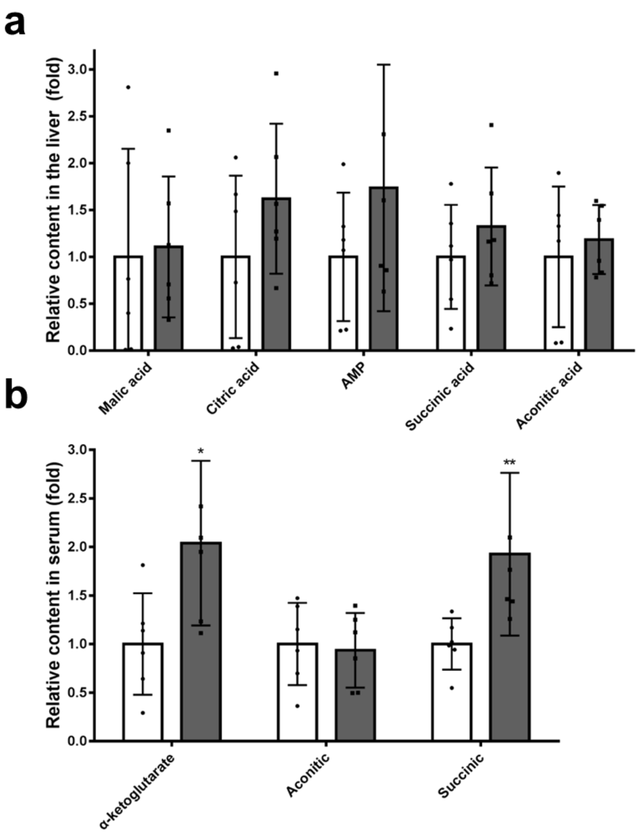

Supplementary Figure 9. Energy metabolomic analysis of liver and serum. (a, b) Liver **(a)** and serum **(b)** energy metabolomic analysis was performed in HuR cKO mice and wild-type littermates fed with HFD for 4 weeks. The energy metabolomic analyses were analyzed. Data are represented as means ± SD from 6 mice (WT, n-6; cKO, n=6); significance was analyzed by using two-tailed Mann-Whitney *U* test (*, $p < 0.05$). All the error bars are equivalent throughout the figure. Source data are provided as a Source Data file. The raw data can be found in the Supplementary Dataset 1.

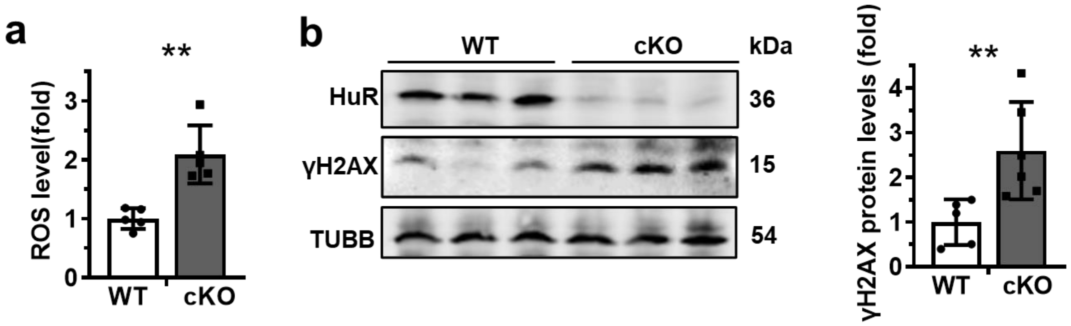

Supplementary Figure 10. Effect of HuR deficiency on the levels of ROS and γH2AX. HuR cKO mice and WT littermates were fed HFD for 4 weeks before analysis. **(a)** ROS levels (WT, n-5; cKO, n=5) were evaluated as described in “Materials and Methods”. Data are the means ± SD from 5 mice (WT, n-5; cKO, n=5); significance was analyzed by using two-tailed Mann-Whitney *U* test (**, $p < 0.01$). **(b)** Lysates prepared from liver were subjected to Western blot analysis to assess the levels of HuR, γH2AX (H2AX), and TUBB. The density of the Western blots for H2AX was represented as means ± SD from 5-6 mice (WT, n-5; cKO, n=6); significance was analyzed by using two-tailed Mann-Whitney *U* test (*, $p < 0.05$； **, $p < 0.01$). All the error bars are equivalent throughout the figure. Source data are provided as a Source Data file.

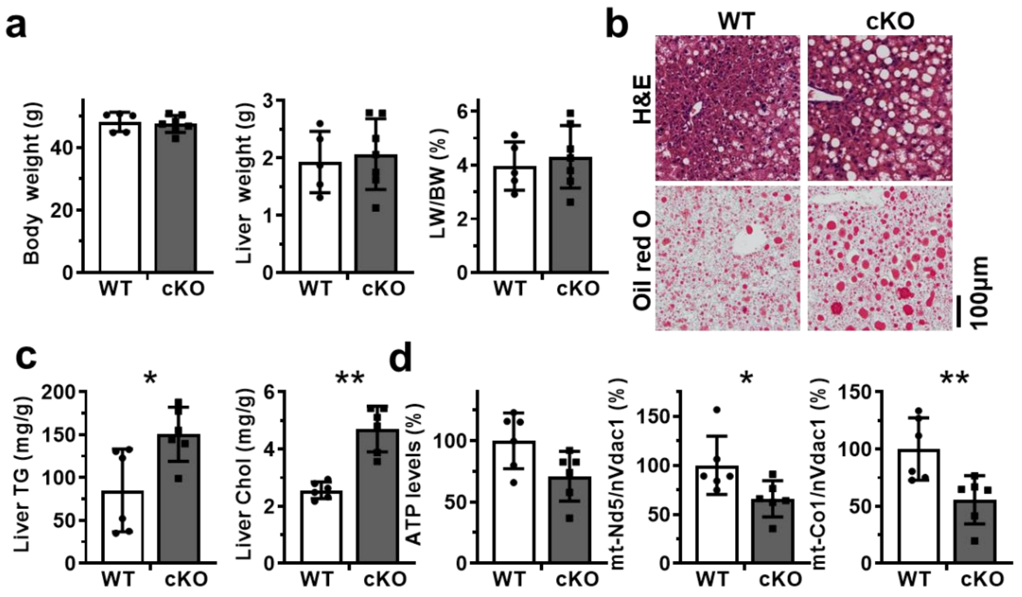

Supplementary Figure 11. Effect of HuR deletion in liver on HFD-induced NAFLD. (a) HuR cKO mice and their WT littermates were fed HFD for 12 weeks. Body weight, liver weight, and the ratio [liver weight/body weight] were analyzed. Data are the means ± SD from 5-7 mice (WT, n=5; cKO, n=7). **(b)** Representative images of staining liver sections with H & E and Oil Red O (WT, n=5; cKO, n=6) to evaluate the phenotype of NAFLD in mice described in (a). **(c)** Mice described in (a) were used for analyzing the levels of liver triglyceride (TG) and Cholesterol (Chol) (WT, n=6; cKO, n=6). **(d)** ATP concentration and relative mtDNA levels were quantified in livers described in (a) (WT, n=6; cKO, n=6). Data in c and d are the means ± SD from 6 mice (WT, n=6; cKO, n=6); significance were analyzed using two-tailed Mann-Whitney *U* test (*, $p < 0.05$; **, $p < 0.01$). All the error bars are equivalent throughout the figure. Source data are provided as a Source Data file.

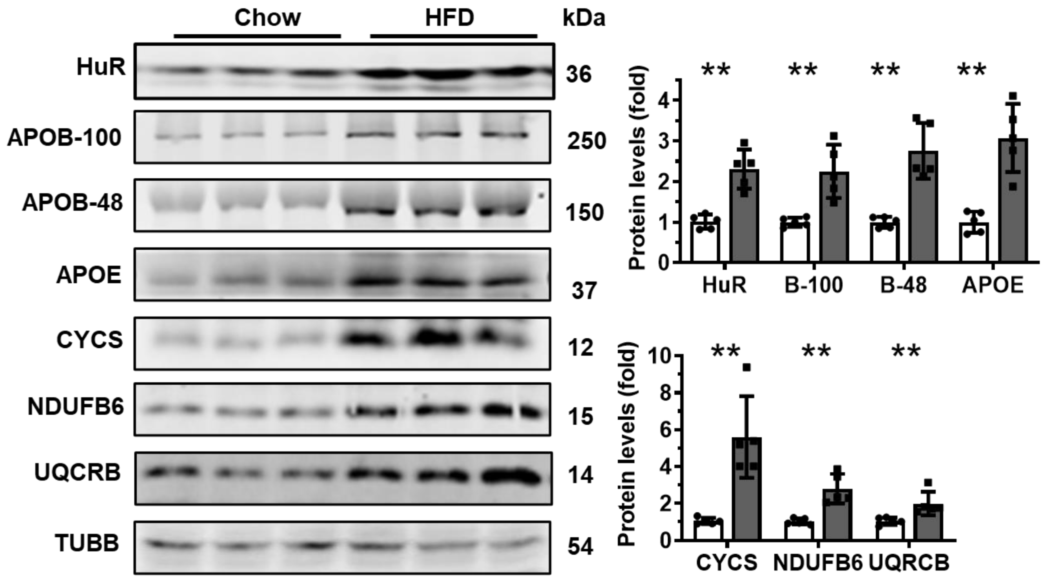

SupplementaryFigure 12. Effect of HFD on the expression of HuR, APOB-100, APOB-48, APOE, CYCS, NDUFB6, and UQCRB.C57BL/6 mice were fed chow diet (n=5) or with HFD (n=5) for 4 weeks, whereupon protein lysates prepared from the livers were subjected to Western blot analysis to test the levels of proteinsHuR, APOB-100, APOB-48, APOE, CYCS, NDUFB6, UQCRB, and TUBB (*left*). Blots are processed from from parallel gels. The density of the signalsfor APOB-100 (B-100), APO-48 (B-48), APOE, CYCS, NDUFB6, and UARCB is represented as means ±SD (Chow, n=5; HFD, n=5); significance is analyzed by using two-tailed Mann-Whitney *U* test (**, $p < 0.01$) (*right*). Blank columns, Chow; Black columns, HFD. All the error bars are equivalent throughout the figure. Source data are provided as a Source Data file.

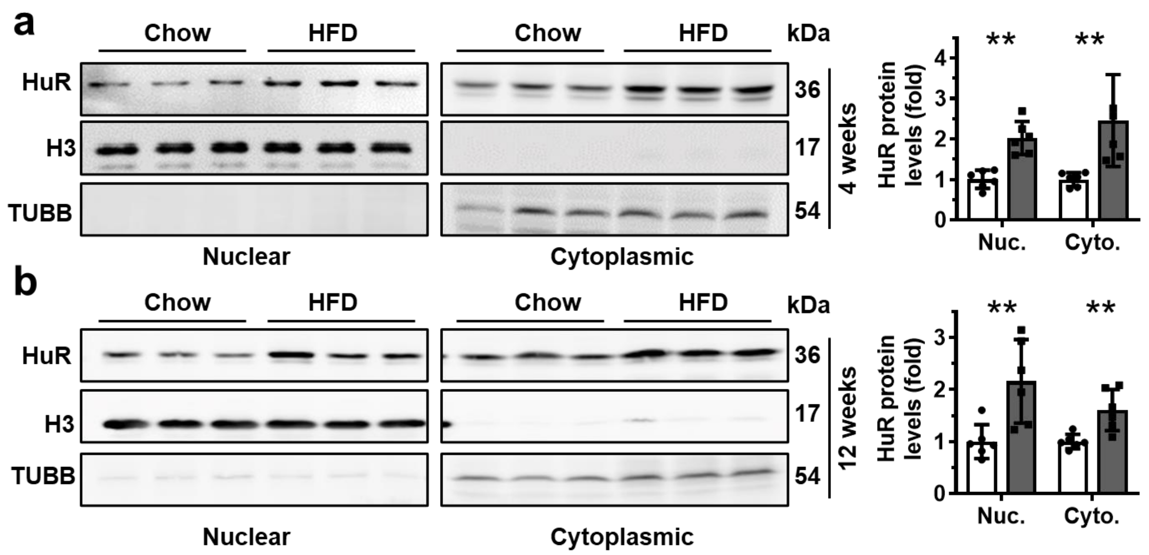

Supplementary Figure 13. Effect of HFD on the levels of HuR in the cytoplasm and nucleus. (a, b) C57BL/6 mice (n=6) were fed with HFD (n=6) for 4 weeks (a) or 12 weeks (b). The cytoplasmic and nuclear protein lysates were prepared from isolated hepatocytes and subjected to Western blot analysis to assess the distribution of HuR in cytoplasm and nucleus. TUBB and H3 were used as loading controls as well as indicators of cytoplasmic (TUBB) and nuclear proteins (H3), respectively. The density of the Western blotting signals for HuR relative to TUBB (for cytoplasm) or H3 (for nucleus) shown in a-b was represented as the means ± SD (n=6); significance was analyzed by two-tailed Mann-Whitney *U* test (*, $p < 0.05$; **, $p < 0.01$). Blank columns, Chow; Black columns, HFD. All the error bars are equivalent throughout the figure. Source data are provided as a Source Data file.

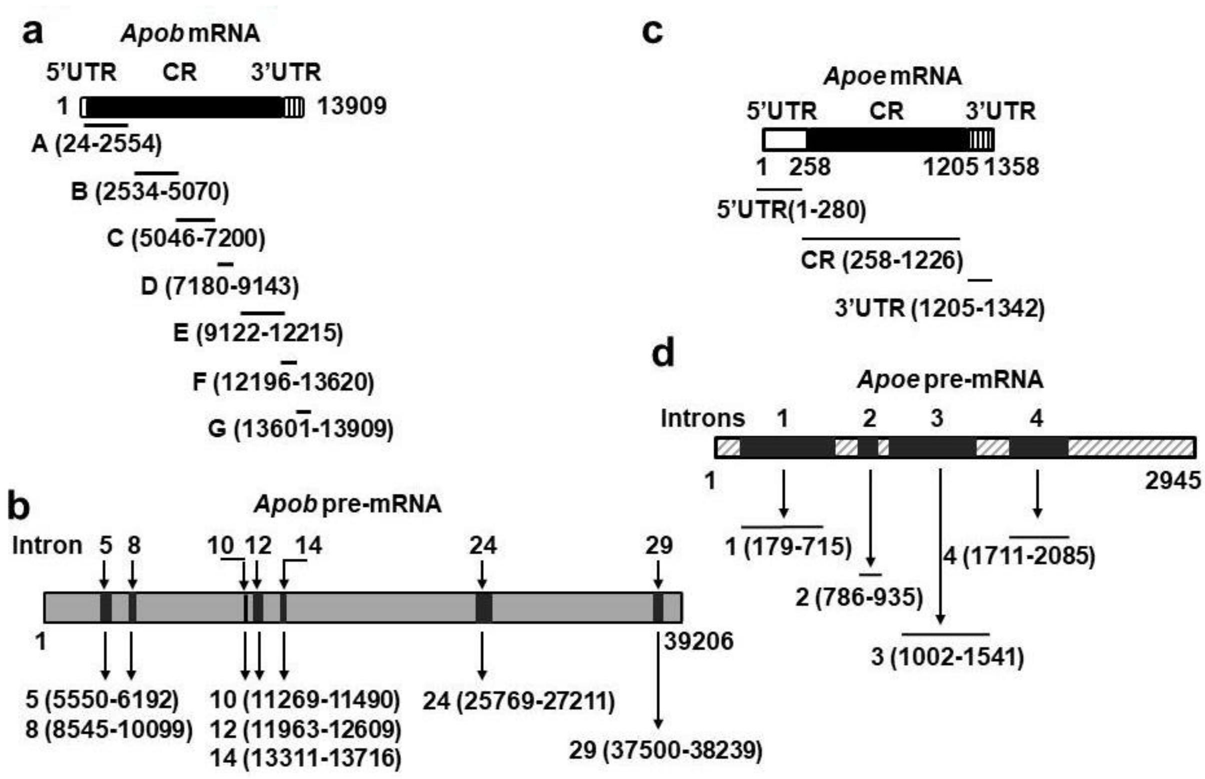

Supplementary Figure 14. Schematic representation of the fragments of mouse *Apob* **(a, b)** and *Apoe* **(c, d)** pre-mRNAs and mRNAs used for RNA pulldown assays. The positions of the fragments are indicated.

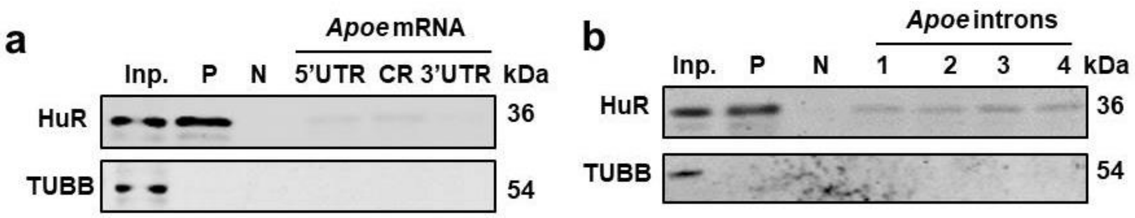

SupplementaryFigure 15. HuR does not associate with the *Apoe* pre-mRNA or the *Apoe* mRNA. (a, b) RNA pulldown assays were performed using Hepa1-6 cell lysates and *in vitro*-transcribed RNAs depicted in Supplementary Figure 12c-d. The presence of HuR in the pulldown materials was assessed by Western blot analysis. *p27* 5'UTR and CR (coding region) served as positive (P) and negative (N) controls, respectively. A 5-µg aliquot input (Inp.) and binding to TUBB were also assessed. Data are representative from 3 independent experiments. Source data are provided as a Source Data file.

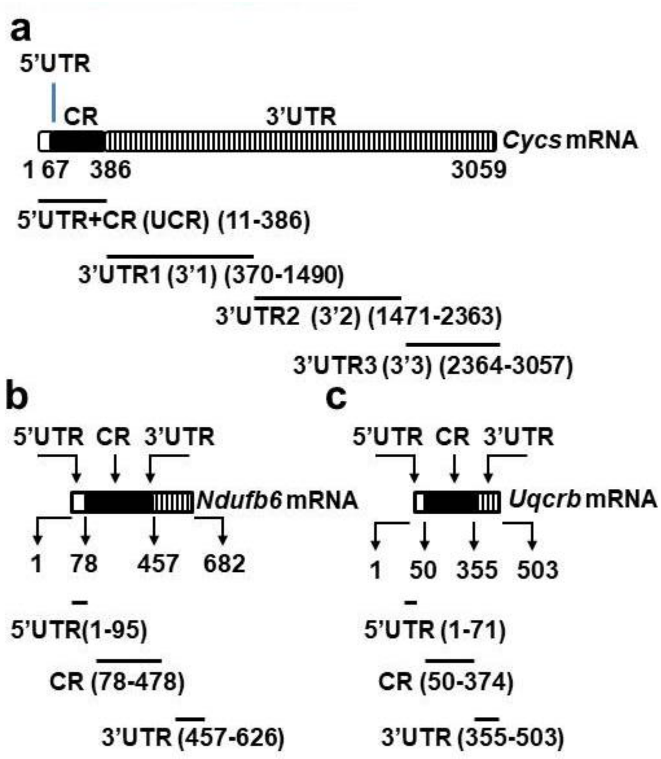

Supplementary Figure 16. (a) Schematic representation of the *CYCS*, *NDUFB6*, and *UQCRB* mRNA fragments used for RNA pulldown assays. The positions of the fragments are indicated. **(b)** Schematic representation depicting the pGL3-derived reporters used for reporter gene analysis.

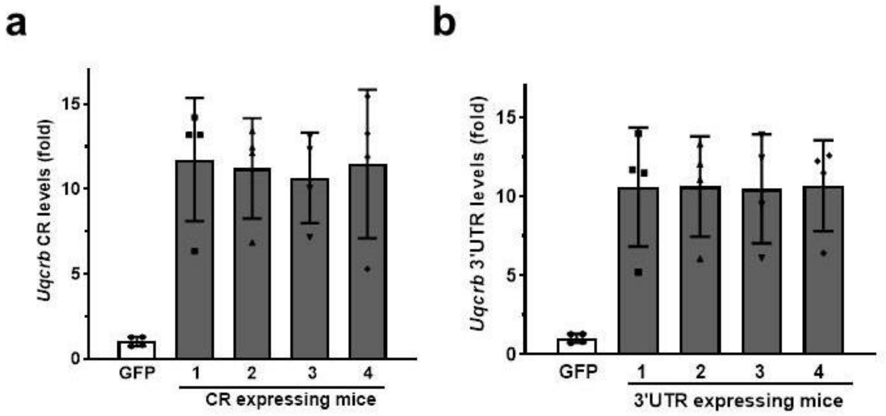

Supplementary Figure 17. Assessment of the expression of *UQCRB* CR and 3'UTR fragments. (a, b) C57BL/6 mice fed with HFD were injected simultaneously with an adeno-associated virus expressing *UQCRB* CR (CR, n=4) or 3'UTR (3'UTR, n=4) fragment. Four weeks later, RNA prepared from livers of each mouse was subjected to real-time qPCR analysis to measure the levels of *UQCRB* CR (a) and 3'UTR (b), respectively. Data for each mouse are the means ± SD from 4 independent experiments (repeats). All the error bars are equivalent throughout the figure. Source data are provided as a Source Data file.

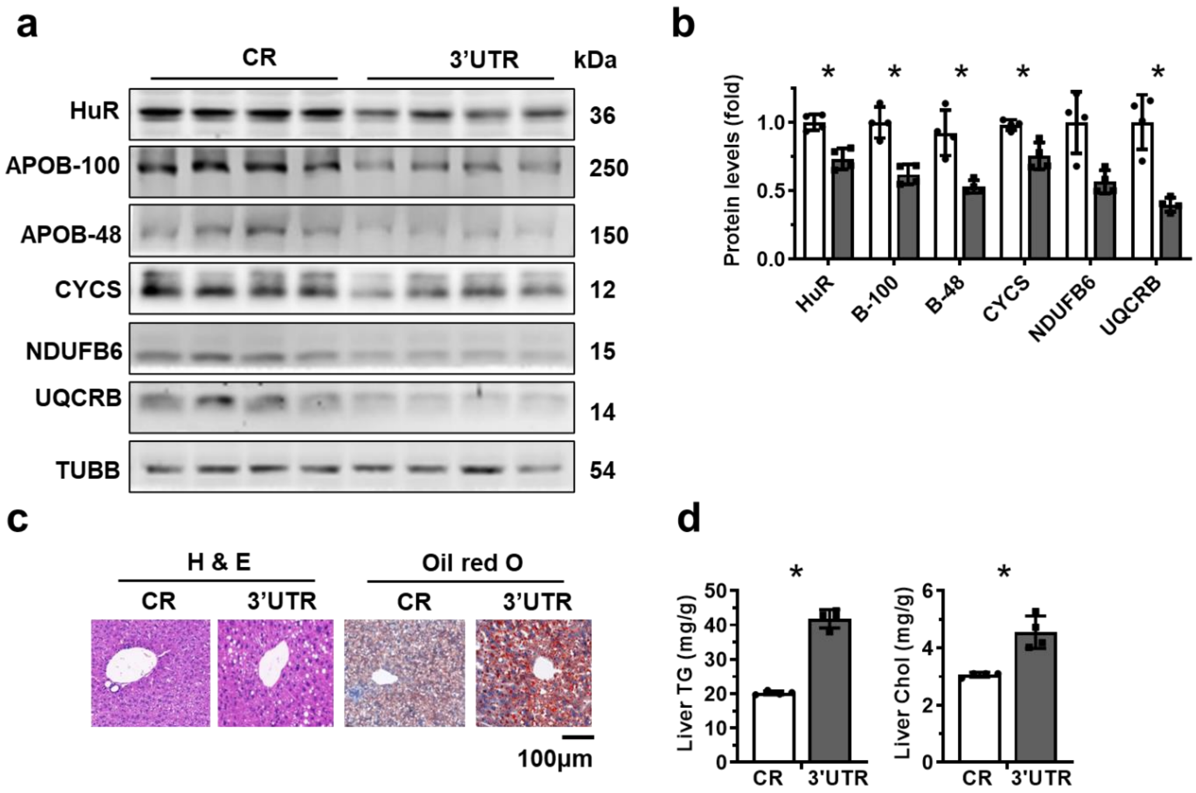

Supplementary Figure 18. Impact of ectopic expression of a *UQCRB* 3'UTR fragment on the effect of HuR on HFD-induced NAFLD. (a) HFD-fed C57BL/6 mice were injected simultaneously with an adeno-associated virus expressing *UQCRB* CR (CR) (n=4) or 3'UTR (3'UTR) fragment (n=4), as described in Supplementary Figure 16. Four weeks later, liver protein lysates were prepared and subjected to Western blot analysis to determine the levels of proteins APOB-100 (B-100), APOB-48 (B-48), CYCS, NDUFB6, UQCRB, and TUBB. Blots are processed from parallel gels. **(b)** The density of the Western blotting signals in (a) are represented as the means ± SD from 4 mice (CR, n=4; 3'UTR, n=4) (Blank columns, CR; Black columns, 3'UTR); significance was analyzed by two-tailed Mann-Whitney *U* test (*, $p < 0.05$). **(c)** Representative images of staining liver sections from mice (CR, n=4; 3'UTR, n=4) described in a using H & E and Oil Red O. **(d)** The levels of liver triglyceride (TG) and cholesterol (Chol) were determined (CR, n=4; 3'UTR, n=4). Data are the means ± SD from 4 mice (CR, n=4; 3'UTR, n=4); significance is analyzed by two-tailed Mann-Whitney *U* test (*, $p < 0.01$). All the error bars are equivalent throughout the figure. Source data are provided as a Source Data file.

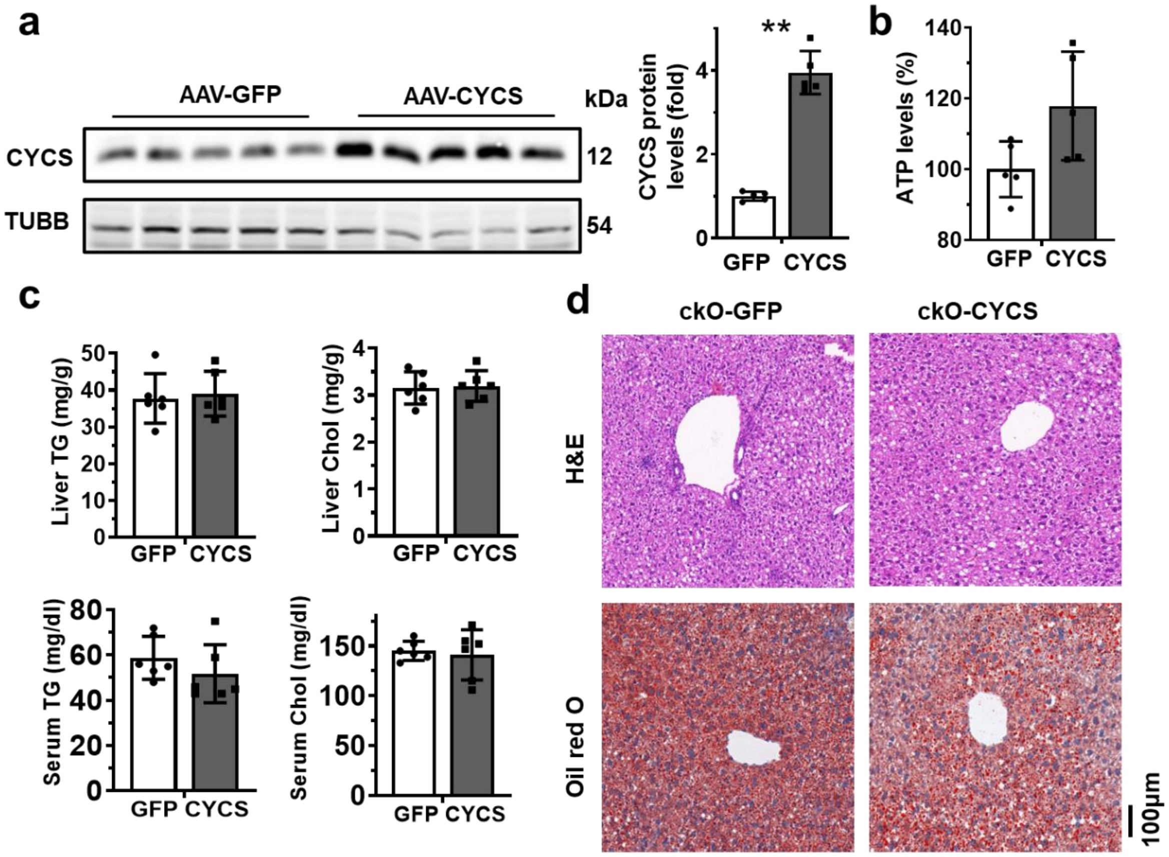

Supplementary Figure 19. Impact of ectopically overexpressing *CYCS* on the effect of HuR cKO in HFD-induced NAFLD. (a) *Left*,HuR cKO mice were infected with an adeno-associated virus expressing CYCS (AAV-CUCS, n=5) or a control virus expressing GFP (AAV-GFP, n=5) and fed with HFD for 4 weeks. The levels of CYCS in the liver tissues were monitored by Western blot analysis. *Right*, the density of the Western blotting signals is represented as the means ± SD from 5 mice; significance was analyzed by two-tailed Mann-Whitney *U* test (**, $p < 0.01$). **(b)** The levels of ATP in tissues described in (a) were analyzed. Data are the means ± SD from 5 mice. **(c)** The levels of liver triglyceride (TG) and cholesterol (Chol) were determined. Data are the means ± SD from 6 mice (AAV-CUCS, n=6; AAV-GFP, n=6). **(d)** Representative images of staining liver sections from mice described in a (AAV-CUCS, n=6; AAV-GFP, n=6) using H & E and Oil Red O. All the error bars are equivalent throughout the figure. Source data are provided as a Source Data file.

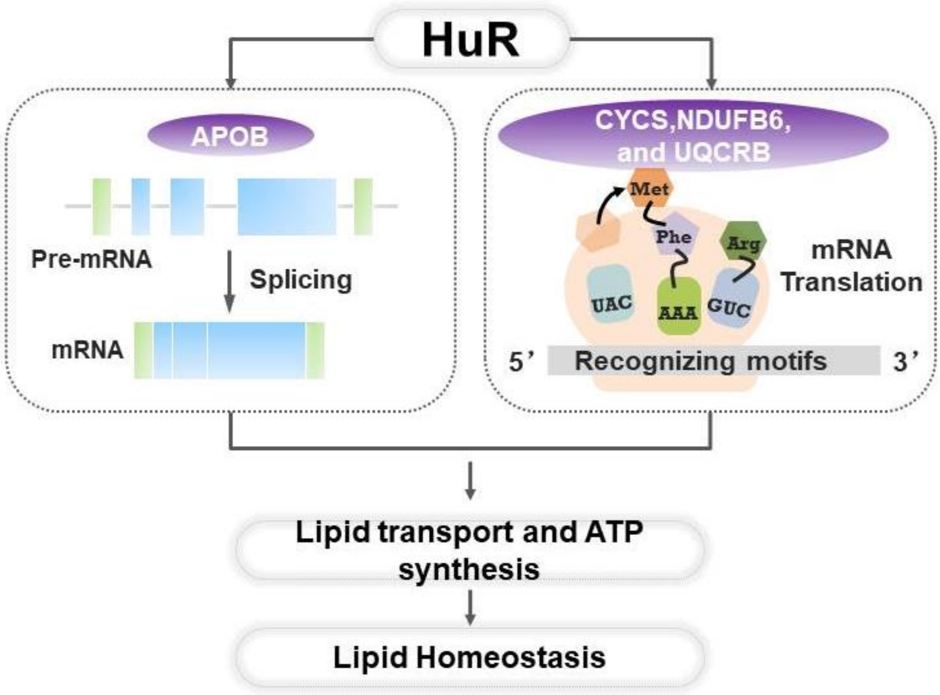

Supplementary Model 1. Model summarizing the major findings in the present study.

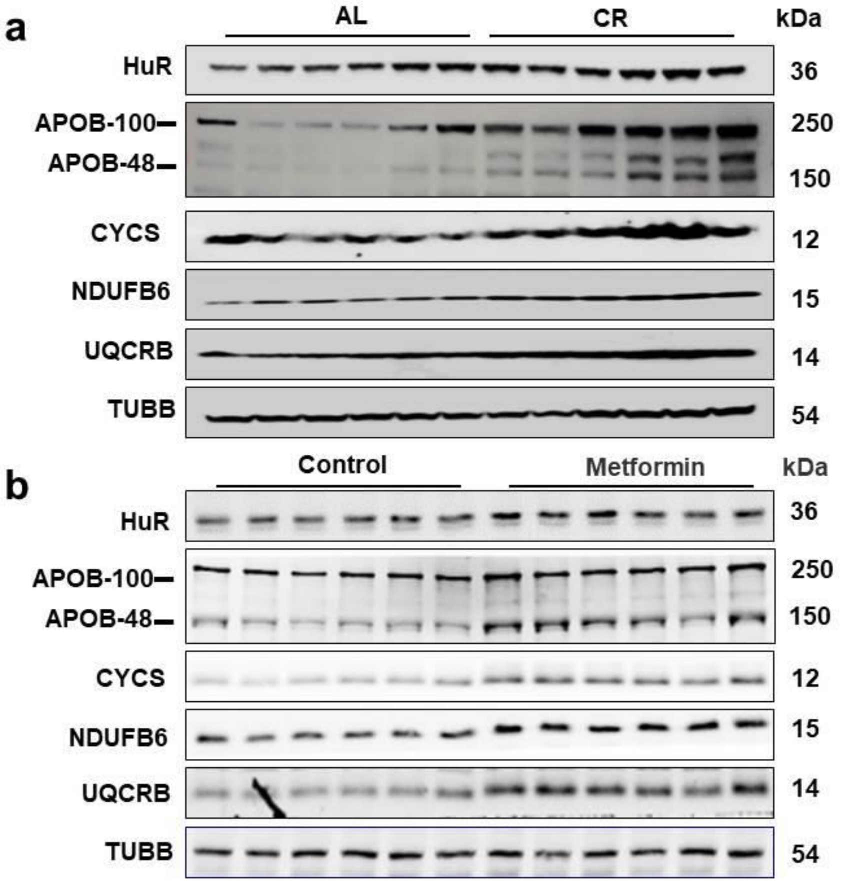

Supplementary Figure 20. Levels of proteins HuR, APOB, CYCS, NDUFB6, and UQCRB in the livers of mice treated with caloric restriction diet and metformin. (a) C57BL/6 mice were fed *ad libitum* (AL) or caloric restriction diet (CR) for 3 months (n=6), whereupon the levels of HuR, APOB-100, APOB-48, CYCSD, NDUFB6, UQCRB, and TUBB in the liver were analyzed by Western blot analysis. Blots were processed from parallel gels. **(b)** C57BL/6mice were orally administrated with metformin (300 mg/kg, 1 time/day) for 3 months (n=6), whereupon the liver tissues were collected and used for Western blot analysis to test the levels of proteins HuR, APOB-100, APOB-48, CYCS, UQCRB, and NDUFB6. Blots in (a) are processed from parallel gels. Source data are provided as a Source Data file.

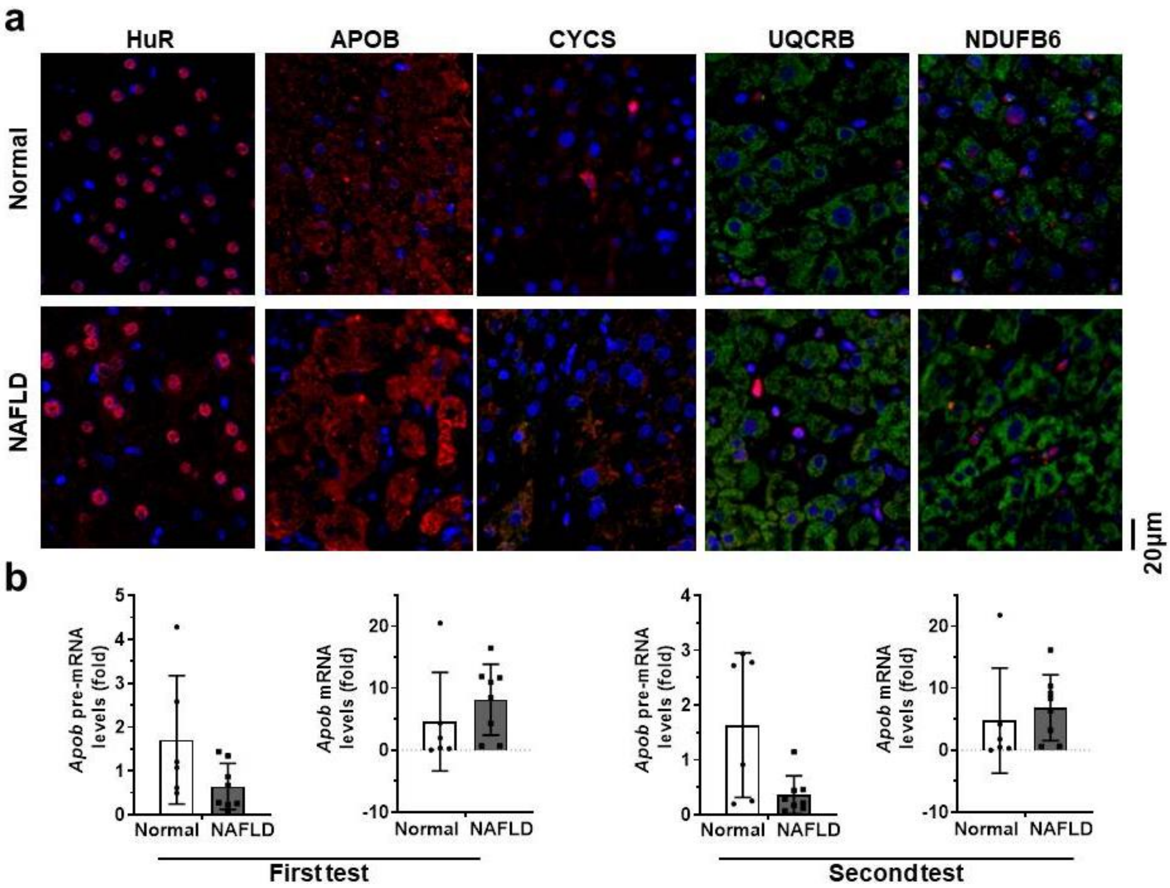

Supplementary Figure 21. Expression levels of HuR, APOB, CYCS, UQCRB, and NDUFB6 in NAFLD patients. (a) Liver slices from NAFLD patients (n=5) and normal human liver (n=5) were subjected to immunohistochemistry analysis to test the protein levels of HuR, APOB, CYCS, UQCRB, and NDUFB6. Data are representative from 5 different samples. **(b)** RNA isolated from NAFLD patients (n=8) and normal human liver tissues (n=6) was subjected to real-time qPCR analysis to test the levels of *Apob* pre-mRNA and mRNA. Data are from 2 repeated tests and the means ± SD of each test is shown. All the error bars are equivalent throughout the figure. Source data are provided as a Source Data file.

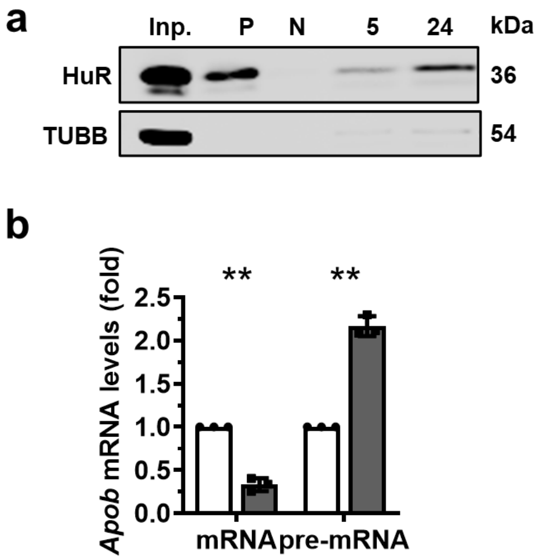

Supplementary Figure 22. HuR may regulate *APOB* mRNA processing in human cells. (a) RNA pulldown assays were performed using HepG2 cell lysates and *in vitro*-transcribed human *APOB* intron 5 (5) and 24 (24) RNA fragments. The presence of HuR in the pulldown materials was assessed by Western blot analysis. *p27* 5'UTR and CR (coding region) served as positive (P) and negative (N) controls, respectively. A 5-μg aliquot input (Inp.) and binding to TUBB were also assessed. Data are representative from 3 independent experiments. **(b)** HepG2 cells were stably transfected with a vector expressing HuR shRNA. RNA was prepared and subjected to RT-qPCR analysis to analyze *APOB* mRNA and pre-mRNA levels. Data are the means ± SD from 3 independent experiments (Blank columns, Control; Black columns, siHuR); significance was analyzed by two-tailed Student's *t* test (**, $p < 0.01$). All the error bars are equivalent throughout the figure. Source data are provided as a Source Data file.

Supplementary reference:

1. Lam SM, Chua GH, Li XJ, Su B, Shui G. Biological relevance of fatty acyl heterogeneity to the neural membrane dynamics of rhesus macaques during normative aging. *Oncotarget***7**, 55970-55989 (2016).

2. Lu J. et al. High-Coverage Targeted Lipidomics Reveals Novel Serum Lipid Predictors and Lipid Pathway Dysregulation Antecedent to Type 2 Diabetes Onset in Normoglycemic Chinese Adults. *Diabetes Care***42**, 2117-2126.(2019).

3. Lam SM, Wang R, Miao H, Li B, Shui G.An integrated method for direct interrogation of sphingolipid homeostasis in the heart and brain tissues of mice through postnatal development up to reproductive senescence. *Anal Chim Acta***1037**, 152-158 (2018).

4. SM Lam et al. Extensive Characterization of Human Tear Fluid Collected Using Different Techniques Unravels the Presence of Novel Lipid Amphiphiles. *J Lipid Res***55**, 289-98 (2014).

5. Li, J.H., Liu, S., Zhou, H., Qu, L.H., &Yang, J.H. starBase v2.0: decoding miRNA-ceRNA, miRNA-ncRNA and protein-RNA interaction networks from large-scale CLIP-Seq data. Nucleic Acids Res (Database issue), D92-7 (2014).
