# Supplementary material for: Hepatic HuR modulates lipid homeostasis in response to high-fat diet
Source: Nat Commun. 2020 Jun 16;11:3067. doi: 10.1038/s41467-020-16918-x (PMC7298042; doi:10.1038/s41467-020-16918-x)
Supplement: Supplementary file 4 — Reporting Summary [file 41467_2020_16918_MOESM4_ESM.pdf]

nature research

Corresponding author(s): Wengong Wang and Zhongzhou Yang

Last updated by author(s): May 23, 2020

# Reporting Summary

Nature Research wishes to improve the reproducibility of the work that we publish. This form provides structure for consistency and transparency in reporting. For further information on Nature Research policies, see Authors & Referees and the Editorial Policy Checklist.

## Statistics

For all statistical analyses, confirm that the following items are present in the figure legend, table legend, main text, or Methods section.

| n/a | Confirmed | |
|---|---|---|
| ☐ | ☒ | The exact sample size (*n*) for each experimental group/condition, given as a discrete number and unit of measurement |
| ☐ | ☒ | A statement on whether measurements were taken from distinct samples or whether the same sample was measured repeatedly |
| ☐ | ☒ | The statistical test(s) used AND whether they are one- or two-sided<br>*Only common tests should be described solely by name; describe more complex techniques in the Methods section.* |
| ☒ | ☐ | A description of all covariates tested |
| ☐ | ☒ | A description of any assumptions or corrections, such as tests of normality and adjustment for multiple comparisons |
| ☐ | ☒ | A full description of the statistical parameters including central tendency (e.g. means) or other basic estimates (e.g. regression coefficient) AND variation (e.g. standard deviation) or associated estimates of uncertainty (e.g. confidence intervals) |
| ☐ | ☒ | For null hypothesis testing, the test statistic (e.g. *F*, *t*, *r*) with confidence intervals, effect sizes, degrees of freedom and *P* value noted<br>*Give P values as exact values whenever suitable.* |
| ☒ | ☐ | For Bayesian analysis, information on the choice of priors and Markov chain Monte Carlo settings |
| ☒ | ☐ | For hierarchical and complex designs, identification of the appropriate level for tests and full reporting of outcomes |
| ☒ | ☐ | Estimates of effect sizes (e.g. Cohen's *d*, Pearson's *r*), indicating how they were calculated |

*Our web collection on statistics for biologists contains articles on many of the points above.*

## Software and code

Policy information about availability of computer code

| | |
|---|---|
| Data collection | Western blot images were acquired on LI-COR Odysssey using Image Studio 5.2 software and analyzed using ImageJ ver 4.0.1;<br>Imaging data for H&E and oil red O staining were collected from NIKON DS-U3 connected to Nikon Eclipse E100;<br>Confocal images were from Zeiss Zen software (version: Zen 2007 light edition);<br>Real-time PCR was performed on a Bio-Rad CFX Connect and the data was captured using BioRad CFX Manager Software version 3.1;<br>Polar lipids were analyzed using an Exion UPLC system coupled with a triple quadrupole/ion trap mass spectrometer (6500 Plus Qtrap; SCIEX) by LipidALL Technologies. Thermo Q-EXACTIVE. A Nexera UPLC (Shimadzu, Kyoto, Japan) system fitted with Q-Exactivequadrupole-Orbitrap mass spectrometer equipped with heated electrospqrayionization (HESI) source (Thermo Fisher Scientific,Waltham, MA, USA) was used to analyze the metabolic profile of both ESI-positive and ESI-negative ion modes. The acquired LC-MS raw data were analyzed by the Progenesis QI software (version 2.3,Waters Corporation Milford, USA).<br>The targets of HuR encoding proteins implicated in ATP synthesis were identified by using the starBase v2.0 website (http://starbase.sysu.edu.cn/index.php46) and KEGG website (https://www.kegg.jp/kegg/).<br>All primers sequences were designed by SnapGene ver 5.0.5. |
| Data analysis | All statistical analysis and P values were obtained using GraphPad Prism ver 8.3.0 and excel 2016. |

For manuscripts utilizing custom algorithms or software that are central to the research but not yet described in published literature, software must be made available to editors/reviewers. We strongly encourage code deposition in a community repository (e.g. GitHub). See the Nature Research guidelines for submitting code & software for further information.

## Data

Policy information about availability of data

All manuscripts must include a data availability statement. This statement should provide the following information, where applicable:

- Accession codes, unique identifiers, or web links for publicly available datasets
- A list of figures that have associated raw data
- A description of any restrictions on data availability

The source data underlying all charts, gels and blots are provided in the Source Data file.

# Field-specific reporting

Please select the one below that is the best fit for your research. If you are not sure, read the appropriate sections before making your selection.

☒ Life sciences ☐ Behavioural & social sciences ☐ Ecological, evolutionary & environmental sciences

For a reference copy of the document with all sections, see nature.com/documents/nr-reporting-summary-flat.pdf

# Life sciences study design

All studies must disclose on these points even when the disclosure is negative.

| | |
|---|---|
| Sample size | No statistical methods were used to determine sample size. Sample sizes was determined to be adequate based on the magnitude and consistency of measurable differences between groups. >= 3 animals or experiments were used as indicated in the figure legends. |
| Data exclusions | No data exclusion |
| Replication | Each experiment was replicated n times (and n is given in each figure for each experiment).<br>All attempts at replicating the observations described in this manuscript were successful. |
| Randomization | No randomization was used in this manuscript. Randomization was not relevant to this study, because we used knockout male mice at similar age (not enough mice for random allocation).The data reported in our experiments is not affected by sample allocation, since the animals are strictly controlled (littermates of same age and gender). |
| Blinding | Blinding was not relevant to this study, as it was a study testing the mechanisms of lipid and ATP metabolism by using mouse model. Data reported for these experiments is not subjective. |

# Reporting for specific materials, systems and methods

We require information from authors about some types of materials, experimental systems and methods used in many studies. Here, indicate whether each material, system or method listed is relevant to your study. If you are not sure if a list item applies to your research, read the appropriate section before selecting a response.

### Materials & experimental systems

| n/a | Involved in the study | |
|---|---|---|
| ☐ | ☒ | Antibodies |
| ☐ | ☒ | Eukaryotic cell lines |
| ☒ | ☐ | Palaeontology |
| ☐ | ☒ | Animals and other organisms |
| ☐ | ☒ | Human research participants |
| ☒ | ☐ | Clinical data |

### Methods

| n/a | Involved in the study | |
|---|---|---|
| ☒ | ☐ | ChIP-seq |
| ☒ | ☐ | Flow cytometry |
| ☒ | ☐ | MRI-based neuroimaging |

## Antibodies

| | |
|---|---|
| Antibodies used | List of antibodies with application and dilutions (IF, immunofluorescence; WB, western blot):<br>polyclonal anti-HuR(WB&IF,1:1000,#11910-1-AP),https://www.ptgcn.com/Products/HuR-Antibody-11910-1-AP.htm<br>polyclonal anti-APOB (WB,1:1000,#20578-1-AP), https://www.ptgcn.com/products/APOB-Antibody-20578-1-AP.htm<br>polyclonal anti-CD36(WB,1:1000,18836-1-AP), https://www.ptgcn.com/Products/CD36-Antibody-18836-1-AP.htm<br>polyclonal anti-FABP1(WB,1:1000,#13626-1-AP), https://www.ptgcn.com/Products/FABP1-Antibody-13626-1-AP.htm<br>polyclonal anti-PPARα(WB,1:1000,#15540-1-AP), https://www.ptgcn.com/Products/PPARA-Antibody-15540-1-AP.htm<br>polyclonal anti-CPT1(WB,1:1000,#15184-1-AP) , https://www.ptgcn.com/Products/CPT1A-Antibody-15184-1-AP.htm<br>polyclonal anti-NDUFB6 (WB&IF,1:1000, #16037-1-AP), https://www.ptgcn.com/Products/NDUFB6-Antibody-16037-1-AP.htm<br>polyclonal anti-UQCRB (WB,1:1000,#10756-1-AP), https://www.ptgcn.com/Products/UQCRB-Antibody-10756-1-AP.htm<br>polyclonal anti-COQ7(WB,1:1000,#15083-1-AP), https://www.ptgcn.com/Products/COQ7-Antibody-15083-1-AP.htm |

polycIonal anti-ATP6V1α (WB,1:1000, #17115-1-AP) ,https://www.ptgcn.com/Products/ATP6V1A-Antibody-17115-1-AP.htm
polyclonal anti-TUBB (WB,1:1000, #10094-1-AP) ,https://www.ptgcn.com/Products/TUBB-Antibody-10094-1-AP.htm
monoclonal anti-CYCS(WB,1:1000,#ab133504),https://www.abcam.cn/cytochrome-c-antibody-epr1327-ab133504.html
monoclonal anti-γH2AX (WB,1:1000,#ab81299),https://www.abcam.cn/gamma-h2ax-phospho-s139-antibody-ep8542y-chip-grade-ab81299.html
polyclonal anti-APOE (WB,1:1000, #AF5178), http://www.affbiotech.com/goods-4485-AF5178-APOE+Antibody.html
polyclonal anti-SCD1 (WB, 1:1000, #DF13253) ,http://www.affbiotech.com/goods-16631-DF13253-SCD1+Antibody.html
polyclonal anti-MTTP (WB,1:1000, #DF6591), http://www.affbiotech.com/goods-5393-DF6591-MTTP+Antibody.html
polyclonal anti-APOB (IF,1:500,#ab20737), https://www.abcam.cn/apolipoprotein-b-antibody-ab20737.html
monoclonal anti-CYCS (IF,1:500,#ab76107), https://www.abcam.cn/cytochrome-c-antibody-ep1326-80-5-ab76107.html
monoclonal anti-UQCRB (IF,1:500,#ab190360), https://www.abcam.cn/uqcrb-antibody-epr15591-ab190360.html

Validation

Validation of each antibody was provided by the manufacturer with each batch as shown in the list above.

# Eukaryotic cell lines

Policy information about cell lines

Cell line source(s)

Mouse hepatoma Hepa1-6 cells were from CCTCC (China Center for Type Culture Collection) and human hepatocellular carcinoma HepG2 cells were from ATCC (American Type Culture Collection)

Authentication

Cell line authentication was ensured by suppliers.

Mycoplasma contamination

We have already performed test for mycoplasma contamination using Myco-Blue Mycoplasma Detector (Vazyme, #D101-01). We did not found contamination issues during the period of this study

Commonly misidentified lines
(See ICLAC register)

None

# Animals and other organisms

Policy information about studies involving animals; ARRIVE guidelines recommended for reporting animal research

Laboratory animals

Species: Mouse (Mus musculus)
Strains: Elavl1 (HuR) floxed mice, Albumin (Alb)-Cre mice and HuR hepatocyte conditional knockout mice (HuR flox/flox). All animals were back-crossed at least 10 generation to a C57Black6/J strain
Sex: males
Age: eight-week-old mice (eight-week-old mice were fed high-fat diet for 4 weeks or 12 weeks.)
Mice were housed in groups of 1-6/cage, at the temperature of 24 °C under a 12 h light-dark cycle with free access to food and water.

Wild animals

None

Field-collected samples

None

Ethics oversight

The animal facility was accredited by the AAALAC (Association for Assessment and Accreditation of Laboratory Animal Care International). All mouse husbandry and experiments were carried out in accordance with the Guide for the Care and Use of Laboratory Animals of the Health Science Center of Peking University, and the IACUC (Institutional Animal Care and Use Committee) of Model Animal Research Center of Nanjing University. Mice were housed in groups with 12-hr dark-light cycles and had free access to food and water.

Note that full information on the approval of the study protocol must also be provided in the manuscript.

# Human research participants

Policy information about studies involving human research participants

Population characteristics

The liver slices and RNA samples from NAFLD patients and normal human liver were very difficult to obtain (only 5-8 samples). They were impossible to be allocated to groups by age, gender, genotypic information, past or current diagnosis, or treatment categories.

Recruitment

Samples were old samples and only allowed for retrospective study . Data from them only could indicate a tendency, as the sample sizes were too small (n=5-8). Informed consent for research and publication was obtained from donors.

Ethics oversight

Ethics were approved by the Research Ethical Committee of Changzhi Medical College (#2019112)

Note that full information on the approval of the study protocol must also be provided in the manuscript.
